# The Moderated Mediation Role of Depressive Symptoms and Physical Health in the Relationship Between Physical Exercise and Sleep Quality Among Emerging Adults

**DOI:** 10.3390/bs16060956

**Published:** 2026-06-10

**Authors:** Lijun Zuo, Guan Yang

**Affiliations:** 1 School of Physical Education, South China University of Technology, Guangzhou 510641, China; 2 Children’s Health and Exercise Research Centre, Department of Public Health and Sport Sciences, University of Exeter, Exeter EX1 2LU, UK; lz446@exeter.ac.uk

**Keywords:** physical exercise, sleep quality, depressive symptoms, physical health, the moderated mediation effect, emerging adults, China family panel studies

## Abstract

Previous research has documented certain associations between physical exercise and sleep quality, yet little is known about the potential influencing mechanisms and boundary conditions underlying them. Thus, the present study aims to examine the potential mediating role of depressive symptoms and the moderating effect of physical health in this relationship. Using the individual-level survey data of 1613 emerging adults aged 18–25 from the China Family Panel Studies (Mage = 20.45 years, SDage = 1.32; 53.8% female), the common method bias test, descriptive statistics and correlation analysis, mediation effect analysis, moderation effect analysis, and simple slope test were sequentially performed using SPSS 27.0, with the significance level set at 5%. The results disclosed that depressive symptoms may play a partial mediating role between physical exercise and sleep quality among emerging adults, and physical health significantly moderated the association between physical exercise and depressive symptoms, indicating a stronger negative association among emerging adults with worse physical health compared to those with better physical health. In addition, exploratory analyses suggested that physical health may also moderate the associations between physical exercise and sleep quality, as well as between depressive symptoms and sleep quality. These findings suggest that emerging adults with lower physical health may often accompany higher depressive symptoms and poorer sleep quality, and also highlight the importance of actively engaging in physical exercise and developing regular exercise habits in daily life to effectively address this problem.

## 1. Introduction

Sleep disturbances have emerged as a major global public health concern alongside accelerating life rhythms and increasing psychosocial stress. Recent epidemiological evidence estimated that approximately 16.2% of adults worldwide experience clinically relevant insomnia, affecting more than 850 million individuals globally (Benjafield et al., 2025). Prevalence estimates vary substantially across countries and regions, with studies from the United States and several European countries generally reporting insomnia prevalence rates ranging from 20% to 30%, while a relatively higher prevalence has been observed in some Asian populations (Benjafield et al., 2025). In China, a recent meta-analysis reported that approximately 19.0% of the general population experience poor sleep quality (P. Chen et al., 2024). In addition, another recent meta-analysis (Spyridonidis et al., 2025) showed that nearly 46.9% of undergraduate university students worldwide experience insomnia symptoms. Compared with working adults, university students are exposed to a distinct set of stressors, including academic demands, adaptation to new living environments, and uncertainty regarding future career trajectories (Gusy et al., 2016; Maymon & Hall, 2021; Pedrelli et al., 2015). As individuals in early adulthood, they must simultaneously meet academic expectations, develop independent living skills, and reconstruct social networks, which collectively increase psychological burden and vulnerability to anxiety and sleep disturbances. In addition, compensatory coping behaviors (Kardefelt-Winther, 2014), such as prolonged use of smartphones or computers before bedtime, may further disrupt sleep through melatonin suppression and irregular sleep–wake schedules.

Sleep plays a fundamental role in physical health, cognitive functioning, and psychological well-being. Insufficient sleep has been associated with impaired attention, memory, and learning efficiency, as well as with anxiety, immune dysfunction (Giri et al., 2024; Lee, 2022), and increased risks of cardiovascular and metabolic diseases (Qi et al., 2025; Palmer et al., 2024). Sleep problems are also a key determinant of quality of life, with long-term insomnia linked to reduced work efficiency, elevated accident risk, and higher rates of depression and suicidal behavior (Bernert et al., 2015; Gardani et al., 2022). Against the backdrop of rapid social and technological change, university students’ lifestyles have become increasingly sedentary. In the present study, regular exercise refers to habitual engagement in physical exercise on a stable and repeated basis, mainly involving moderate-intensity aerobic and recreational activities such as brisk walking, dancing, bowling, table tennis, and badminton. Unlike continuous indicators of exercise duration or intensity, the concept of regular exercise habit emphasizes behavioral consistency and lifestyle integration, reflecting whether individuals routinely incorporate exercise into their daily lives. From a behavioral health perspective, maintaining a regular exercise habit may represent a relatively stable self-regulatory behavior associated with healthier daily routines, better stress management, and more adaptive lifestyle patterns, all of which may contribute to improved sleep outcomes. Therefore, insufficient engagement in regular physical exercise has become a salient health concern among university students.

A growing number of studies demonstrate that inadequate physical exercise not only compromises physical health but also adversely affects psychological well-being and cognitive functioning (Smith & Merwin, 2021; Zhang & Jiang, 2023). Low levels of exercise are associated with increased risks of obesity, cardiovascular disease, anxiety, and depressive symptoms, whereas regular physical exercise has been shown to enhance cognitive performance and promote psychological relaxation (Kanaley et al., 2022; Jeong et al., 2019; S. Wang et al., 2025). Importantly, maintaining a regular exercise habit is also associated with improved sleep quality, potentially through physiological mechanisms (e.g., improved sleep architecture) and psychosocial pathways (e.g., stress reduction and decreased maladaptive behaviors such as excessive smartphone use) (J. Wang et al., 2024). However, the existing studies have largely focused on direct associations or single mediating mechanisms, offering limited insight into the conditions under which regular exercise habits influence sleep outcomes (Kovacevic et al., 2018; Xie et al., 2024). Specifically, this study aims to examine a moderated mediation model in which regular physical exercise influences sleep quality both directly and indirectly through depressive symptoms, while physical health moderates these pathways.

### 1.1. The Mediating Role of Depressive Symptoms

Psychological mechanisms such as mastery experiences and attentional distraction suggest that exercise may reduce depressive symptoms by providing distraction from negative thoughts while simultaneously enhancing perceptions of mastery, self-efficacy, self-worth, and environmental control, which are consistently associated with improved emotional well-being (Gordon et al., 2018; Pedersen & Saltin, 2015; Md Zemberi et al., 2020; Noetel et al., 2024). The endorphin hypothesis further suggests that exercise stimulates the release of β-endorphins, which are associated with improved mood and reduced pain perception. In addition, the monoamine hypothesis proposes that exercise increases neurotransmitters such as dopamine, serotonin, and norepinephrine, which improve mood and exert antidepressant effects (Craft & Perna, 2004). Depressive symptoms are closely associated with sleep-related problems, including difficulty initiating sleep and sleep disturbances, whereas lower levels of depression are generally linked to better sleep quality, indicating that depression may substantially impair sleep-related functioning (World Health Organization, 2025; Micah et al., 2021).

Empirical evidence has increasingly demonstrated a consistent negative association between physical exercise and depressive symptoms across diverse populations. Studies among university students and general populations show that regular physical exercise reduces depressive symptoms and lowers the risk of depression (Pedersen & Saltin, 2015; Md Zemberi et al., 2020). This further indicates that physical exercise has moderate-to-strong antidepressant effects in both clinical and non-clinical populations, and even low-intensity activities, such as 150 min of walking per week, confer protective effects. Within the sleep health framework and neuroplasticity theories, physical exercise may indirectly improve sleep quality by alleviating depressive symptoms, highlighting depression as a key mechanism linking exercise and sleep outcomes.

### 1.2. The Moderating Role of Physical Health

Physical exercise has long been regarded as an important contributor to both physical and psychological health, a view reflected in national fitness policies in China as well as in a large body of empirical research (Herbert, 2022; Garber et al., 2011; Zhang & Jiang, 2023). From a physiological standpoint, exercise primarily involves skeletal muscle contraction, which plays a key role in maintaining normal bodily function. Experimental studies (Z. Chen et al., 2021) have shown that contracting muscle cells can directly suppress pro-inflammatory responses, providing biological evidence for the close link between physical exercise and physical health. Together, these findings support the notion that regular physical exercise is beneficial for overall health status.

Physical health is also closely related to sleep quality. Individuals with better physical functioning and emotional well-being are more likely to experience stable and restorative sleep. In addition to its physiological effects, physical exercise helps relieve stress, promotes social interaction, and supports emotional regulation, all of which contribute to improved health outcomes. Empirical studies among university students consistently indicate that higher levels of physical exercise are associated with better physical health and fewer negative psychological symptoms. However, the existing evidence suggests that the relationship between physical exercise and sleep quality may not be entirely direct, as declines in physical or psychological health can impair sleep, even among individuals who exercise regularly. This highlights physical health as an important mediating factor in the association between physical exercise and sleep quality.

### 1.3. The Present Study

**Figure 1:**
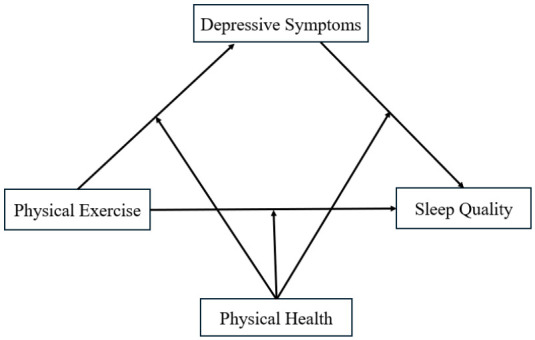
The hypothesized moderated mediation model.

Given the factors mentioned above, physical exercise has been consistently shown to be beneficial for both physical and mental health across diverse populations, and is also associated with improved sleep quality. In addition, depressive symptoms have been identified as a key psychological factor influencing sleep quality, while physical health represents an important physiological indicator that may shape individuals’ responses to physical exercise. Drawing on the conditional process framework proposed by Hayes (Hayes, 2013), this study develops a moderated mediation model to examine the internal mechanisms and boundary conditions underlying the association between physical exercise and sleep quality. As illustrated in Figure 1, physical exercise is hypothesized to influence sleep quality both directly and indirectly through depressive symptoms. In this model, depressive symptoms are proposed as a mediator, while physical health is expected to moderate the effect of physical exercise on depressive symptoms, as well as the direct effect of physical exercise on sleep quality and the relationship between depressive symptoms and sleep quality.

Accordingly, the following hypotheses are proposed: physical exercise is positively associated with sleep quality (H1); depressive symptoms may mediate the relationship between physical exercise and sleep quality (H2); and physical health may moderate the pathways within this mediation process (H3). The hypothesized moderated mediation model is presented in Figure 1.

## 2. Methods

### 2.1. Procedure and Participants

The present cross-sectional study aims to examine the underlying associations and mechanisms between physical exercise and sleep quality among emerging adults using data from the China Family Panel Studies (CFPS), a nationally representative longitudinal survey conducted by the Institute of Social Science Survey at Peking University. The CFPS has been implemented biennially since 2010 and collects comprehensive information at the individual, household, and community levels. For the present study, data from the 2022 CFPS wave (released in 2025) were accessed and downloaded from the official CFPS database following standard user registration procedures. The dataset covers 25 provinces, autonomous regions, and municipalities in China, with an initial sample of approximately 16,000 households and 28,530 respondents.

The data screening procedure was conducted as follows. First, only respondents aged 18–25 years were extracted from the full dataset. Second, cases with missing values on key study variables, including physical exercise, sleep quality, and relevant covariates, were excluded using listwise deletion. Third, the remaining valid cases were retained for analysis. After these procedures, a final analytical sample of 1613 participants was obtained. Among them, 53.8% were female. The mean age was 20.45 years (SD = 1.32). Regarding educational level, 59.1% had completed junior college education, while 40.9% held a bachelor’s degree. In terms of household registration, 57.7% were from rural areas and 42.3% were from urban areas.

### 2.2. Measurements

#### 2.2.1. Physical Exercise

The independent variable in this study was physical exercise, which was used as a dichotomous variable indicating whether participants had a regular exercise habit. Physical exercise was assessed using three items measuring exercise frequency, duration, and intensity. Exercise frequency was assessed by asking how often participants engaged in physical health or leisure activities during the past 12 months; exercise duration referred to the usual length of each exercise session; and exercise intensity captured participants’ perceived physiological response during exercise. Following established criteria in prior research (Hu et al., 2020) participants were classified as regular exercisers (coded as 1) if they met all three conditions: exercising more than three times per week, engaging in sessions lasting longer than 30 min, and reaching at least a moderate level of exercise intensity. Participants who did not meet these criteria were classified as non-regular exercisers (coded as 0). Accordingly, physical exercise was treated as a binary variable in the subsequent analyses.

#### 2.2.2. Sleep Quality

Sleep quality was selected as the dependent variable in this study. Specifically, self-rated sleep quality reported by respondents was used as a proxy for overall sleep quality. In the 2022 CFPS questionnaire, sleep quality was assessed using the item “I sleep poorly.” Responses were recorded on a four-point Likert scale ranging from 1 to 4, with the following options: “rarely (less than one day),” “sometimes (1–2 days),” “often (3–4 days),” and “most of the time (5–7 days).” In the original dataset, higher scores indicated poorer sleep quality. To ensure consistency in interpretation across variables, the sleep quality item was reverse-coded in the present study so that higher scores represent better sleep quality. All descriptive statistics, correlation analyses, and regression models reported in this study were based on the recoded variable.

#### 2.2.3. Depressive Symptoms

Depressive symptoms was treated as the mediating variable and then assessed using the 8-item Center for Epidemiological Studies Depression Scale (CESD-8). Participants were asked to evaluate the frequency of eight depressive symptoms based on their experiences during the past week, and response options included “rarely or none of the time”, “some of the time”, “occasionally”, and “most of the time”, corresponding to scores from 1 to 4, respectively. The eight items covered the following symptoms: (1) feeling depressed; (2) feeling happy; (3) feeling lonely; (4) enjoying life; (5) feeling sad; (6) feeling that everything requires effort; (7) experiencing insomnia; and (8) feeling that life is difficult to continue. It should be noted that two items—“feeling happy” and “enjoying life”—were reverse-coded, such that higher scores on these items indicate lower levels of depressive symptoms. The total CESD-8 score ranged from 8 to 32, with higher scores indicating more severe depressive symptoms. The CESD-8 has been widely used for self-assessment in the general population, and the Chinese version has demonstrated good validity and reliability (Missinne et al., 2014). In this work, this scale exhibited high internal consistency, with a Cronbach’s α of 0.843.

#### 2.2.4. Physical Health

Physical health was selected as the moderating variable in the present study, and then measured through the CFPS 2022 questionnaire item, “How would you rate your overall physical health?” Respondents rated their health status based on subjective perception, with response options ranging from “very healthy” to “unhealthy”. The variable was coded on a scale from 1 to 5, with higher values indicating better physical health.

#### 2.2.5. Demographic Variables

To more accurately estimate the effect of physical exercise on sleep quality among emerging adults and to minimize potential confounding effects, the current work reasonably selected several typical control variables in accordance with the prior literature (Gong et al., 2024; S. Wang et al., 2025), which includes age, gender, educational level, and residence status. Specifically speaking, gender was coded as female = 0 and male = 1; educational level was coded as junior college or below = 0 and bachelor’s degree or above = 1; and residence status was coded as rural registration = 0 and urban registration = 1 in this research.

### 2.3. Statistical Analysis

The screened data in this work were compiled and organized using Epidata version 3.1, and all subsequent statistical analyses were conducted using Stata 17.0 and SPSS 27.0. Continuous variables were summarized as mean and standard deviations (M ± SD), and a two-tailed significance level of *p* < 0.05 was adopted for all statistical tests. The analytical procedures were executed in the following sequence: (1) Harman’s single-factor test was performed to assess the potential presence of common method bias in the self-reported data. (2) Pearson’s correlation analysis was used to examine the bivariate associations between sleep quality, depressive symptoms, and physical health. (3) The mediating role of depressive symptoms was tested using Model 4 of the PROCESS macro. Bias-corrected bootstrap procedures with 5000 resampling were applied to estimate the total effect, direct effect, and indirect effect, along with their corresponding 95% confidence intervals (CIs). (4) The moderating effect of physical health was examined using Model 59 of the PROCESS macro. Simple slope analysis was subsequently conducted to visualize the moderation effect, and also to illustrate how the relationship between physical exercise and sleep quality varied across different levels of physical health.

## 3. Results

### 3.1. Common Method Bias Test

Due to the self-reported measures employed in this research, this approach could lead to common method bias, potentially affecting the observed relationships between variables and compromising the accuracy of the findings. Given this, the Harman’s single-factor test was conducted to assess the presence of common method bias in this study. The results indicated that four factors had eigenvalues greater than 1, and the first unrotated factor accounted for 29.73% of the total variance, which is below the commonly accepted threshold of 40% (Podsakoff et al., 2003). These findings suggest that common method bias is unlikely to be a serious concern in the present study.

### 3.2. Descriptive Statistics and Correlation Analyses

Pearson’s correlation analysis revealed significant associations among all variables (Table 1). Sleep quality was positively correlated with physical health (*p* < 0.001), but was negatively associated with depression symptoms (*p* < 0.001). Moreover, physical health was significantly and negatively related to depression symptoms (*p* < 0.001). Taken together, these findings indicate robust relationships among main variables, and also provide a sound empirical basis for subsequent moderated mediation analysis.

### 3.3. Mediation Effect Test

To test H2, the mediating effect of depressive symptoms on the relationship between physical exercise and sleep quality among emerging adults was examined using Model 4 of the SPSS PROCESS macro. As shown in Table 2, after controlling for age, sex, residence status, and educational level, physical exercise significantly negatively predicted depression (*b* = −1.131, *p* < 0.001) and also significantly positively predicted sleep quality (*b* = 0.235, *p* < 0.001). Depressive symptoms, in turn, were a significant negative predictor of sleep quality (*b* = −0.137, *p* < 0.001). It can be easily seen that when depressive symptoms were included as a mediating variable, the positive predictive effect of physical exercise on sleep quality remained significant (*b* = 0.080, *p* = 0.032), indicating that depressive symptoms partially mediate the relationship between physical exercise and sleep quality. To further validate the mediating effect, a bootstrap analysis was properly conducted. As shown in Table 3, the 95% bootstrap confidence interval for the indirect path from physical exercise to sleep quality via depressive symptoms did not include zero (−0.204 to −0.107), indicating a significant mediating effect of depression. The indirect effect size was 0.155, accounting for 65.95% of the total effect. After controlling for the mediating variable, the direct effect of physical exercise on sleep quality remained significant, with the 95% confidence interval not including zero, suggesting that depressive symptoms partially mediate the relationship between physical exercise and sleep quality. It is clear that these findings provide further support for the proposed mediation model.

### 3.4. Moderation Effect Test

To examine whether the mediating effect of depressive symptoms in the relationship between physical exercise and sleep quality is influenced by other variables, physical health was introduced as a moderator in a moderated mediation analysis. As shown in Table 4, after controlling for age, sex, educational level, and residence status, physical exercise significantly negatively predicted depressive symptoms (*b* = −0.928, *p* < 0.001), while physical health also significantly negatively predicted depressive symptoms (*b* = −0.936, *p* < 0.001). In Model 2, depressive symptoms significantly negatively predicted sleep quality (*b* = −0.131, *p* < 0.001), whereas physical health significantly positively predicted sleep quality (*b* = 0.076, *p* < 0.001). However, the direct effect of physical exercise on sleep quality was not significant (*b* = 0.072, *p* > 0.05). Additionally, physical health did not significantly moderate the relationship between depressive symptoms and sleep quality (*b* = −0.001, *p* > 0.05). Moreover, the interaction between physical exercise and physical health significantly predicted depressive symptoms (*b* = 0.588, *p* = 0.002), whereas the interaction between physical exercise and physical health did not significantly predict sleep quality (*b* = 0.071, *p* > 0.05). These results indicate that physical health moderates the association between physical exercise and depressive symptoms, but does not significantly moderate the relationship between depressive symptoms and sleep quality or the direct association between physical exercise and sleep quality.

**Figure 2:**
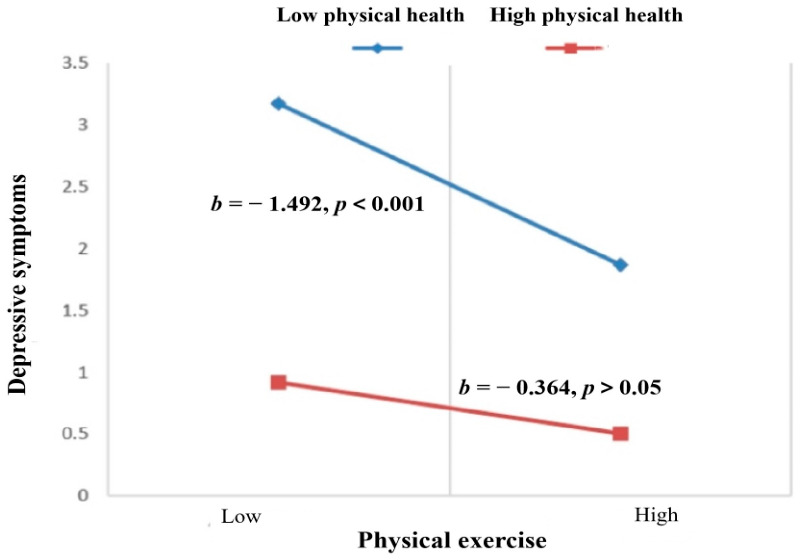
Moderating effect of physical health on the association between physical exercise and depressive symptoms.

To further examine the moderating effect of physical health, a simple slope analysis was conducted, and the moderated mediation effects are also depicted in Figure 2. In the present study, “low” and “high” levels of physical health were operationalized as one standard deviation below and above the mean, respectively. The simple slope analysis indicated that at low levels of physical health, physical exercise was significantly negatively associated with depressive symptoms (*b* = −1.492, *p* < 0.001). In contrast, at high levels of physical health, the association between physical exercise and depressive symptoms remained negative but was not statistically significant (*b* = −0.364, *p* > 0.05), suggesting that the association was weaker at higher levels of physical health.

## 4. Discussion

The present study contributes to the growing literature on sleep health among emerging adults by examining the indirect association between physical exercise and sleep quality through depressive symptoms, as well as the moderating role of physical health. The findings indicate that physical exercise was indirectly associated with better sleep quality through reduced depressive symptoms. In addition, physical health significantly moderated the relationship between physical exercise and depressive symptoms, whereas no significant moderating effects were observed in the pathways involving sleep quality. Together, these findings provide additional insight into the psychological and health-related mechanisms underlying sleep quality in this population.

Consistent with prior evidence, this study demonstrates a significant positive association between physical exercise and sleep quality among emerging adults, supporting H1. Higher levels of physical exercise were associated with better sleep quality, reinforcing the well-established role of physical exercise as a protective health behavior (Bandura, 2004; Darko et al., 2015). The existing research (Kredlow et al., 2015) has shown that exercise is associated with improved sleep efficiency, shortened sleep onset latency, and increased total sleep duration, associations that have been linked to physiological processes such as melatonin secretion (Carlson et al., 2019) and thermoregulation (Périard et al., 2021). Nevertheless, the “dosage” of physical exercise—specifically its timing—may act as a double-edged sword for nocturnal rest. While our findings highlight a general positive association, it is worth noting that vigorous activity performed too close to bedtime may temporarily increase sympathetic nervous system activity and core body temperature, potentially leading to sleep onset difficulties for some individuals. This nuance suggests that the relationship between exercise and sleep is not a simple linear progression. Notably, the beneficial associations between physical exercise and sleep quality varied across subgroups. Female students experienced more pronounced improvements, which may reflect greater psychological and social benefits associated with exercise, including stress reduction and enhanced social support (Kruk et al., 2021; Alnawwar et al., 2023). Differences were also observed by educational level and household registration status, suggesting that the association between physical exercise and sleep quality may be shaped by broader social and structural contexts, such as types of stress exposure and access to health-promoting resources (Golaszewski et al., 2022). Together, these findings highlight that while physical exercise broadly is associated with sleep quality, its association is not uniform across populations. Interventions aiming to improve sleep among emerging adults should therefore consider gender- and context-sensitive approaches to maximize their public health impact.

The present study demonstrates that depressive symptoms play a significant mediating role in the association between physical exercise and sleep quality among emerging adults, supporting H2. This finding supports the hypothesis that the sleep-promoting effects of physical exercise are partially transmitted through improvements in mental health. Several theoretical perspectives help explain this mediating pathway (Igartua & Hayes, 2021). From a psychological standpoint, physical exercise may alleviate depressive symptoms by reducing stress, decreasing rumination, and enhancing emotional regulation. Exercise also serves as an adaptive coping strategy that allows individuals to disengage from daily stressors and negative affect, thereby improving psychological well-being (Fang et al., 2019). These changes may subsequently create more favorable conditions for sleep initiation and maintenance, given the well-established link between depressive symptoms and sleep disturbances. From a physiological perspective, exercise-induced changes, such as increased serotonin availability and enhanced neuroplasticity (De Sousa Fernandes et al., 2020; Corrêa et al., 2025), may further contribute to reductions in depressive symptoms, indirectly benefiting sleep quality. Together, these psychological and biological mechanisms provide a coherent explanation for how physical exercise influences sleep through depressive symptoms. Importantly, the mediating role of depressive symptoms underscores the interconnected nature of mental health and sleep health in the populations of emerging adults. Rather than acting solely through direct physiological pathways, physical exercise appears to improve sleep quality by first alleviating depressive symptoms. This finding highlights depressive symptoms as a key intervention target within exercise-based health promotion programs. Interventions that simultaneously promote physical exercise and address depressive symptoms may therefore be particularly effective in improving sleep quality among emerging adults.

The findings partially supported H3. Specifically, physical health significantly moderated the association between physical exercise and depressive symptoms, whereas no significant moderating effects were found in the relationships between physical exercise and sleep quality or between depressive symptoms and sleep quality. These findings suggest that physical health may mainly influence the emotional benefits associated with physical exercise, rather than directly altering sleep-related outcomes. More specifically, the association between physical exercise and depressive symptoms was stronger among students with poorer physical health, but weaker and non-significant among those with better physical health. One possible explanation is that individuals with poorer physical health may rely more heavily on exercise as a coping resource for emotional regulation. In contrast, emerging adults with better physical health may already possess relatively stable emotional functioning, making the additional mental health benefits associated with exercise less pronounced. This pattern may reflect a “ceiling effect” or the principle of diminishing marginal utility in health promotion (Duncan & Murnane, 2010; Lawton & Fujiwara, 2016). In contrast, physical health did not significantly moderate the relationships between physical exercise and sleep quality or between depressive symptoms and sleep quality. One possible explanation is that the association between physical exercise and sleep quality may operate through relatively universal physiological mechanisms, such as circadian rhythm regulation, energy expenditure, and physical fatigue, which may function similarly across different levels of physical health. In addition, sleep quality is influenced by multiple factors, including academic stress, daily lifestyle habits, environmental conditions, and screen exposure, which may weaken the moderating role of physical health. Similarly, although depressive symptoms are closely associated with sleep disturbances, this relationship may remain relatively stable regardless of baseline physical health status, resulting in a non-significant effect.

Lastly, although the present study identified the mediating role of depressive symptoms and the moderating role of physical health, the underlying mechanisms require further investigation through longitudinal and experimental research. Future studies should consider additional factors associated with sleep quality, such as dietary habits, screen exposure, and social support. Moreover, different forms and intensities of physical exercise may show distinct associations with depressive symptoms and sleep quality, and therefore warrant further examination. It is also important to note that potential bidirectional relationships may exist among physical exercise, depressive symptoms, and sleep quality. Although the present findings suggest that physical exercise was indirectly associated with sleep quality through depressive symptoms, poor sleep quality and depressive symptoms may also reduce motivation for exercise participation. Therefore, future longitudinal and cross-lagged studies are needed to further clarify the reciprocal relationships among these variables.

## 5. Practical Implications

Taken together, the findings of this study highlight the necessity of adopting an integrated perspective when addressing sleep problems among emerging adults, rather than viewing sleep quality solely as a physiological outcome. The results underscore the interconnected roles among physical exercise, depressive symptoms, and physical health in shaping sleep quality. This integrated framework contributes to the existing literature by elucidating a conditional process through which physical exercise is associated with sleep quality, thereby advancing a more nuanced understanding of the behavioral and psychological mechanisms underlying sleep health in populations of emerging adults. Moreover, from a practical perspective, the findings suggest that interventions aimed at improving sleep quality among emerging adults may benefit from simultaneously promoting regular physical exercise and addressing mental health conditions, particularly depressive symptoms. Physical exercise promotion alone may be insufficient if psychological distress and physical health status are not taken into account, but tailored health promotion strategies that combine exercise-based interventions with mental health screening and support may therefore be more effective in improving sleep quality. In the Chinese context, where emerging adults often face high academic and career-related pressures, integrating physical exercise programs into university health services and public health initiatives could be regarded as a highly feasible and beneficial approach. More broadly, the evidence provided by the present study also offers implications for the development of holistic health promotion strategies targeting emerging adults, not only in China but also in other countries experiencing similar social and developmental challenges.

## 6. Limitations and Future Directions

Except for those positive implications mentioned above, several underlying limitations of the present study should also be objectively acknowledged. At first, the cross-sectional design restricts causal inference among main variables in this research. Although the observed associations are theoretically grounded, longitudinal studies or experimental means are needed to examine temporal ordering and causal pathways. Future research employing prospective cohort designs could provide stronger evidence for the dynamic relationships among these variables. Secondly, the study relied primarily on self-reported measures, which may be subject to recall bias and social desirability effects. Although validated instruments were used, future studies would benefit from incorporating objective assessments, such as wearable devices, polysomnography, or physiological indicators (e.g., heart rate variability), to enhance measurement accuracy and robustness. Thirdly, the study did not differentiate between specific exercise types. Given the heterogeneity of exercise modalities and their potentially distinct effects on mental health and sleep outcomes, future research should compare different forms of physical exercise, including aerobic, resistance, and other flexibility-based exercise. Such evidence would help inform more precise and individualized exercise recommendations for emerging adults. Fourthly, even though depressive symptoms and physical health were examined as key psychological and physical factors in this work, other variables may also play important mediating or moderating roles between physical exercise and sleep quality. Given that, future research should consider incorporating broader psychosocial factors, such as stress, resilience, social support, and lifestyle behaviors, using advanced analytical approaches to develop a more comprehensive understanding of the mechanisms linking physical exercise and sleep quality. Finally, while the present study employed simple slope analyses to probe the moderating effect of physical health, future research may further apply Johnson–Neyman techniques to provide a more fine-grained depiction of the region of significance for the interaction effect, thereby allowing for a more continuous interpretation of moderation patterns.

## 7. Conclusions

The present study aimed to clarify the underlying relationships among physical exercise, depressive symptoms, physical health, and sleep quality among emerging adults. Particular attention was given to examining whether depressive symptoms mediate the association between physical exercise and sleep quality and whether physical health moderates this indirect relationship. The study yielded three main findings. First, physical exercise was positively associated with sleep quality, suggesting that higher levels of physical exercise are linked to better sleep outcomes among emerging adults. Second, depressive symptoms were found to mediate the relationship between physical exercise and sleep quality, indicating that the beneficial association between physical exercise and sleep quality may partially operate through reduced depressive symptoms. Third, physical health was shown to moderate the indirect association between physical exercise and sleep quality via depressive symptoms, with the mediating effect appearing to be stronger among individuals with poorer physical health. In essence, the present study suggests that the interplay among physical exercise, depressive symptoms, and physical health may play an important role in shaping sleep quality among emerging adults. These findings highlight the potential importance of promoting physical exercise not only for its direct association with sleep quality, but also for its possible role in alleviating depressive symptoms, particularly among emerging adults with relatively poor physical health. Nevertheless, these conclusions should be interpreted with caution due to the cross-sectional nature of the study. Future research employing longitudinal and experimental designs is needed to further examine these associations and to better understand the psychological and physical mechanisms linking physical exercise to sleep quality.

## Figures and Tables

**Table 1:** Descriptive statistics and correlation analysis among major variables.

Variable	M ± SD	1	2	3
1. Sleep quality	1.66 ± 0.83	1		
2. Physical health	3.34 ± 0.95	0.199 ***	1	
3. Depressive symptoms	12.81 ± 3.49	−0.593 ***	−0.216 ***	1

M = mean score; SD = standard deviation. *** *p* < 0.001.

**Table 2:** Mediation analysis of depression between physical exercise and sleep quality.

Predictor	Model 1: Depressive Symptoms		Model 2: Sleep Quality		Model 3: Sleep Quality	
	*b*	*t*	*b*	*t*	*b*	*t*
Constant	14.31	35.43 ***	1.913	19.65	−0.053	−0.496
Age	−0.025	−2.665 **	0.002	0.684	−0.002	−1.014
Sex	−0.217	−1.238	0.090	2.136 *	0.060	1.739
Residence status	−0.275	−1.398	0.084	1.780	0.047	1.195
Educational level	−0.118	−0.667	0.079	1.859	0.064	1.799
Physical exercise	−1.131	−6.057 ***	0.235	5.233 ***	0.080	2.140 *
Depressive symptoms					−0.137	−27.81 ***
*R* ^2^	0.032		0.025		0.342	
*F*	10.84 ***		8.335 ***		139.31 ***	

*b* = non-standardized regression coefficient. * *p* < 0.05, ** *p* < 0.01, *** *p* < 0.001.

**Table 3:** Bootstrap analysis of the mediating effect between physical exercise and sleep quality.

Pathway	Effect	SE	*t*	LLCI	ULCI
Total effect	0.235	0.045	5.233 ***	0.147	0.281
Direct effect (physical exercise → sleep quality)	0.080	0.037	2.141 *	0.006	0.095
Indirect effect (physical exercise → depressive symptoms → sleep quality)	0.155	0.041	−3.781 ***	−0.204	−0.107

SE = standard error, LLCI = lower-level CI, ULCI = upper-level CI. * *p* < 0.05, *** *p* < 0.001.

**Table 4:** Testing for the moderating effect of physical health.

Predictor	Model 1: Depressive Symptoms			Model 2: Sleep Quality		
	*b*	*SE*	*t*	*b*	*SE*	*t*
Constant	1.553	0.393	3.952 ***	1.717	0.081	21.27 ***
Age	−0.041	0.009	−4.425 ***	0.001	0.002	0.275
Sex	−0.102	0.169	−0.604	−0.053	0.035	−1.522
Residence status	−0.206	0.190	−1.082	−0.043	0.039	−1.097
Educational level	−0.133	0.171	−0.775	−0.066	0.035	−1.885
Physical exercise	−0.928	0.182	−5.096 ***	0.072	0.038	1.928
Physical health	−0.936	0.089	−10.53 ***	0.076	0.019	4.020 ***
Depressive symptoms				−0.131	0.005	−25.15 ***
Physical exercise × physical health	0.588	0.190	3.097 **	0.071	0.040	1.779
Depressive symptoms × physical health				−0.001	0.004	−0.947
*R* ^2^	0.103			0.350		
*F*	26.42 ***			96.09 ***		

*b* = non-standardized regression coefficient, SE = standard error, ** *p* < 0.01, *** *p* < 0.001.

## Data Availability

The datasets used and/or analyzed during the current study are available from the corresponding author on reasonable request.
